# Description of *Hoplolaimus bachlongviensis* sp. n. (Nematoda: Hoplolaimidae) from banana soil in Vietnam

**DOI:** 10.3897/BDJ.3.e6523

**Published:** 2015-11-10

**Authors:** Tien Huu Nguyen, Quang Duc Bui, Phap Quang Trinh

**Affiliations:** ‡ Institute of Ecology and Biological Resources, Vietnam Academy of Science and Technology, Hanoi, Vietnam

**Keywords:** bananas, *
Hoplolaimus
*, new species, Tonkin Gulf, Vietnam

## Abstract

**Background:**

The genus *Hoplolaimus* Daday, 1905 belongs to the subfamily Hoplolaimine Filipiev, 1934 of family Hoplolaimidae Filipiev, 1934 (Krall 1990). Daday established this genus on a single female of *H. tylenchiformis* recovered from a mud hole on Banco Island, Paraguay in 1905 (Sher 1963​, Krall 1990). *Hoplolaimus* species are distributed worldwide and cause damage on numerous agricultural crops (Luc et al. 1990 Robbins et al. 1998​). In 1992, Handoo and Golden reviewed 29 valid species of genus *Hoplolaimus* Dayday, 1905 (Handoo and Golden 1992). Siddiqi (2000) recognised three subgenera in *Hoplolaimus*: *Hoplolaimus (Hoplolaimus)* with ten species, is characterized by lateral field distinct, with four incisures, excretory pore behind hemizonid; *Hoplolaimus (Basirolaimus)* with 18 species, is characterized by lateral field with one to three incisures, obliterated, excretory pore anterior to hemizonid, dorsal oesophageal gland quadrinucleate; and *Hoplolaimus (Ethiolaimus)* with four species is characterized by lateral field with one to three incisures, obliterated; excretory pore anterior to hemizonid, dorsal oesophageal gland uninucleate (Siddiqi 2000). Since then, *Hoplolaimus puriensis* Ali, Shaheen & Pervez, 2009 has been described (​Ali et al. 2009). Up to now, there have been two species of genus *Hoplolaimus* reported in Vietnam, viz *H. seinhorsti* and *H. chambus* (Nguyen and Nguyen 2000).

**New information:**

*Hoplolaimus bachlongviensis* sp. n. was isolated from banana soil in Bach Long Vi Island, Vietnam. The female of this species is described and illustrated below. Some diagnostic characters of this species include body slightly curved ventrally, offset lip region exhibiting three to four annules, lateral field reduced, pharyngeal glands with six nuclei, excretory pore anterior to hemizonid, epiptygma absent, intestine not overlapping rectum and male was not found.

## Introduction

In many surveys of plant parasitic nematodes on bananas in agriculture and natural forest systems in mainland of Vietnam, only two species *Hoplolaimus seinhorsti* Luc, 1958 and *H. chambus* Jairajpuri & Baqri, 1973 were recorded (Nguyen and Nguyen 2000). During a survey of plant parasitic nematodes in Bach Long Vi Island (located about 130 km off the mainland of Vietnam), a *Hoplolaimus* sp. was collected which was morphologically different from other known species. Herein this species is morphologically characterised and described as *Hoplolaimus bachlongviensis* sp. n.

## Materials and methods

The nematodes were detected from banana soil samples in Bach Long Vi Island, Vietnam (20°07'52.8" N, 107°43'56.6" E). Soil nematodes were extracted using the decanting and modified Baermann tray method (Whitehead and Hemming 1965). Measurements were made on permanent slides of heat-killed nematodes with fixative TAF and ethanol-glycerin dehydration according to the method described by Seinhorst (1959) and modified bySeinhorst (1959), De Grisse (1969). For morphological examination, nematodes were observed through the Olympus BX-51 light microscope, and photographed with an Olympus U-TV 0.5xC-3 digital camera.

## Taxon treatments

### Hoplolaimus bachlongviensis sp. n.

urn:lsid:zoobank.org:act:2632ABBD-A056-4AAA-B1CC-DB668A2EED07

#### Materials

- **Type status:** Holotype. **Occurrence:** sex: female; behavior: migratory ectoparasite on banana roots; **Taxon:** family: Hoplolaimidae; genus: Hoplolaimus; **Location:** island: Bach Long Vi; stateProvince: Hai Phong; county: Vietnam; verbatimCoordinates: 20°07'52.8"N, 107°43'56.6"E; **Identification:** identificationID: BLV-4050-1
- **Type status:** Paratype. **Occurrence:** sex: female; behavior: migratory ectoparasite on banana roots; **Taxon:** family: Hoplolaimidae; genus: Hoplolaimus; **Location:** island: Bach Long Vi; stateProvince: Hai Phong; county: Vietnam; verbatimCoordinates: 20°07'52.8"N, 107°43'56.6"E; **Identification:** identificationID: BLV-4050-2

#### Description

*Females*

(Table 1; Figs 1, 2, 3)

Body slightly curved ventrally, rarely C-shaped, cylinder, vermiform, tapering slightly at both ends. Lip region offset, usually bearing 4 distinct annuli, sometimes 3 annuli, basal ring of lip region with 6 longitudinal striations (Figs 1a, 3a, b). Cuticular annulation prominent. Lateral field reduced and represented by the interruption of body annuli as a single incisures, but often indistinct (Figs 1e, 3d). Stylet large and strong with prominent tulip-shaped basal knob represented by three anterior projection, DGO about 4 µm behind spear base (Figs 1a, 3a). Metacorpus ovate with well-developed, sclerotized valve. Pharyngeal glands with 6 nuclei (Fig. 3a). Distinct nerve ring encircling isthmus. Excretory pore situated within range from level of nerve ring to level of esophago-intestineal valve or even somewhat more posterior. Hemizonid distinct large, two annules in length, located about seven annules behind Excretory pore (Figs 1b, 3a). Hemizonion located 8-10 annules posterior to hemizonid. Phasmids (scutella) anterior and posterior to vulva, large and conspicuous (Figs 1e, 3d). Vulva prominent, transverse slit at mid-body; epiptygma absent (Figs 1c, d, 3c). Ovaries two, outstretched (amphidelphic), spermatheca empty (Fig. 2a). Intestine not overlapping rectum (Figs 1f, 3e, f). Tail short, rounded, shorter than the anal body diameter, usually with 9-13 annuli (Figs 1f, 2b, 3e, f).

#### Diagnosis

*Hoplolaimus bachlongviensis* sp. n. is characterized by lip region set off, lateral field reduced, represented by a single incisure on the body, but often indistinct, Pharyngeal glands with six nuclei, excretory pore prominent and located seven annules anterior to hemizonid, epiptygma absent, intestine not overlapping rectum, male absent.

#### Etymology

The species is named after the geographic location, Bach Long Vi Island of Vietnam.

#### Notes

*Males*: Unknown

#### Type material

Female holotype and seven female paratypes deposited in the nematode collection of the Institute of Ecology and Biological Resources, Vietnam Academy of Science and Technology, 18 Hoang Quoc Viet str., Hanoi, Vietnam. Accession numbers: IEBR.Nema4050-1 (one female hoplotype); IEBR.Nema4050-2 (8 female paratypes).

## Discussion

*Hoplolaimus bachlongviensis* sp. n. is similar to *Hoplolaimus seinhorsti*, *H. chambus*, *H. columbus* Sher, 1963 and *H. pararobustus* (Schuurmans Stekhoven & Teunissen, 1938) Sher, 1963 by having excretory pore anterior to hemizonid, lateral field reduced, represented by interruptions of annules as a single incisure, often indistinct, pharyngeal glands with six nuclei (Handoo and Golden 1992). However, *H. bachlongviensis* sp. n. differs from *H. seinhorsti* by epiptygma absent *vs* present and number of longitudinal striations on basal ring 6 *vs* 8-12. It differs from *H. chambus* by male absent *vs* present, epiptygma absent *vs* present, intestine not overlapping rectum *vs* overlapping rectum. *Hoplolaimus bachlongviensis* sp. n. differs from *H. columbus* in having fewer tail annuli 9-13 *vs* 16-22; a=22-27 *vs* a=30-38; b=6-8 *vs* b=9.1-12.4; DGO=3-6 *vs* DGO=9-13; epiptygma absent *vs* present; hemizonid located about 7 annuli behind excretory pore *vs* 2-5 annuli and intestine not overlapping rectum *vs* overlapping rectum. *Hoplolaimus bachlongviensis* sp. n. differs from *H. pararobustus* by male absent *vs* present, intestine not overlapping rectum *vs* overlapping rectum, epiptygma absent *vs* present and sperm absent *vs* present.

*Hoplolaimus bachlongviensis* sp. n. is distinguished from *H. sheri* Suryawanshi 1971 by having lateral field reduced and represented by the interruption of body annuli as a single incisures *vs* two incisures in lateral field; having longer stylet 44-50 *vs* 40-45; having fewer longitudinal striations on basal ring 6 *vs* 20; hemizonid is conspicuous *vs* obscure; a=22-27 *vs* a=26-30; b=6-8 *vs* b=9.7-11.5.

*Hoplolaimus bachlongviensis* sp. n. differs from *H. puriensis* by lateral field reduced, represented by a single incisure on the body, but often indistinct *vs* four lateral lines, longer stylet 44-50 µm *vs* shorter stylet 32-35 µm.

## Supplementary Material

XML Treatment for Hoplolaimus bachlongviensis

## Figures and Tables

**Figure 1a.**
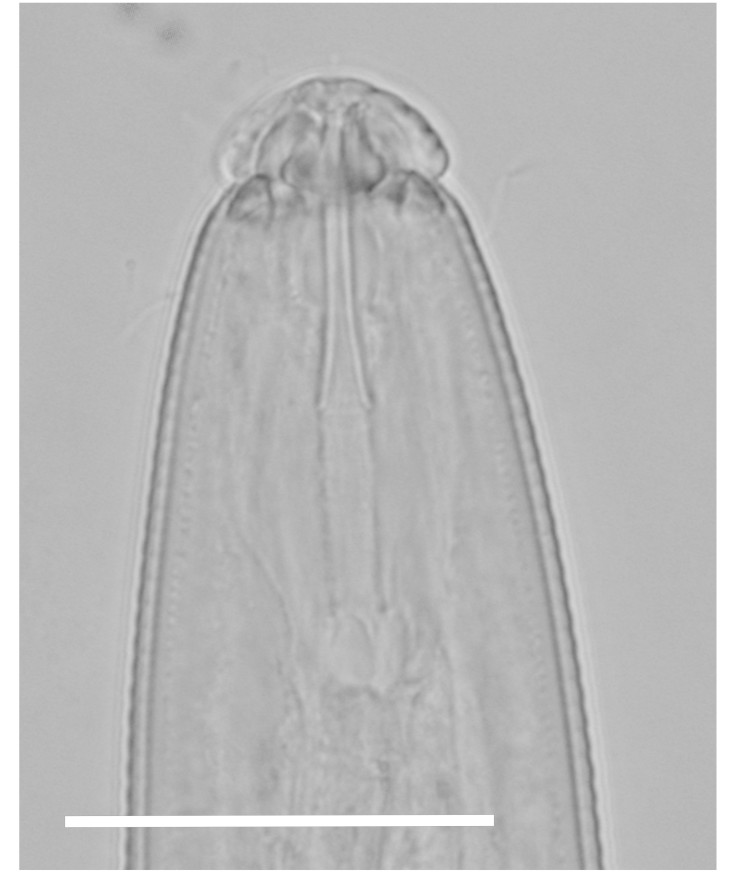
Anterior end

**Figure 1b.**
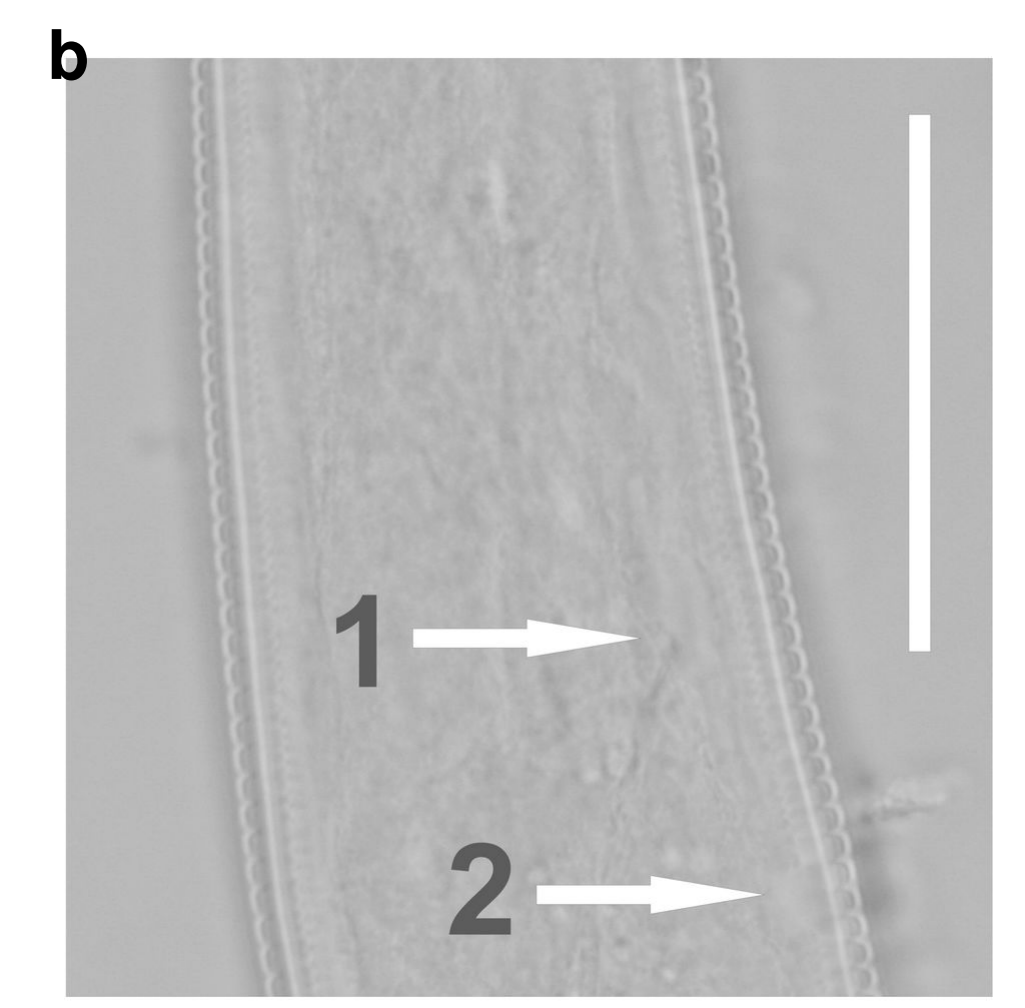
Excretory pore (arrow 1) and hemizonid (arrow 2)

**Figure 1c.**
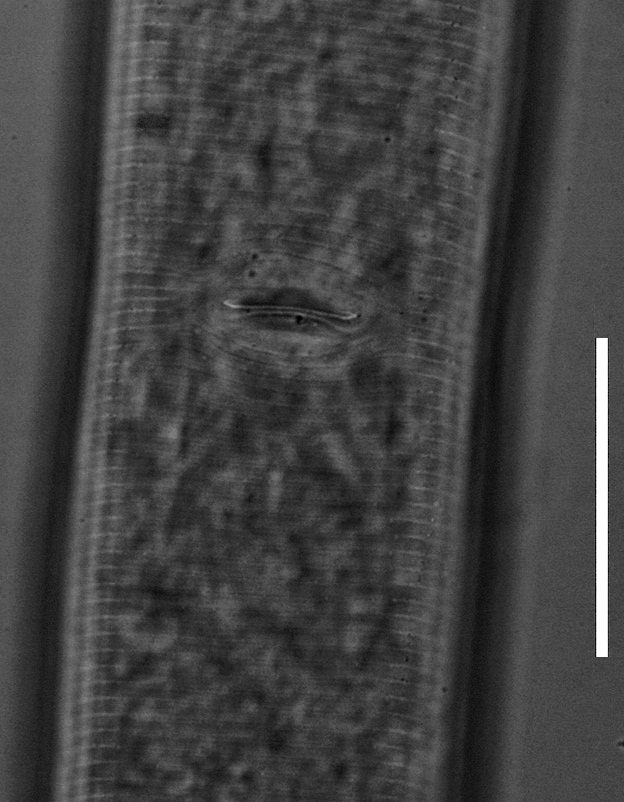
Vulva region in ventral view

**Figure 1d.**
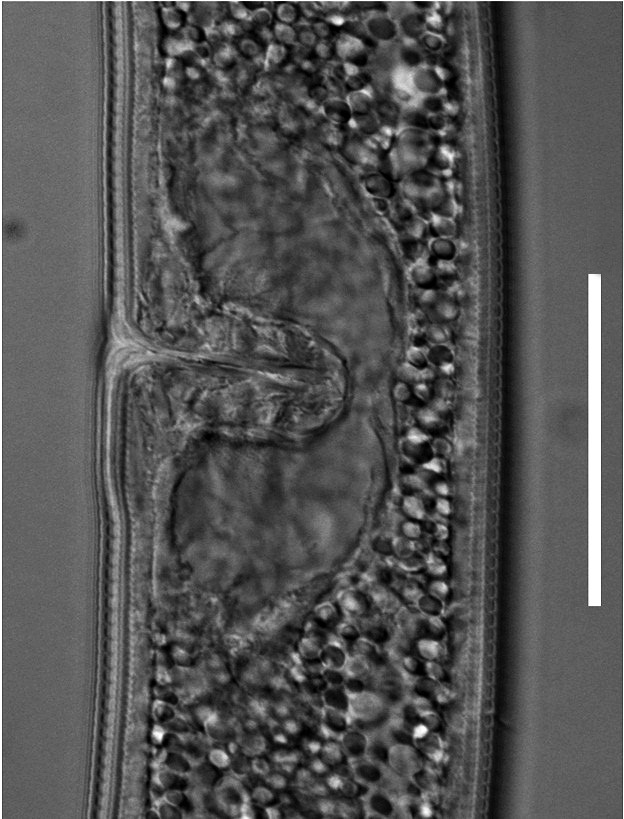
Vulva region in lateral view

**Figure 1e.**
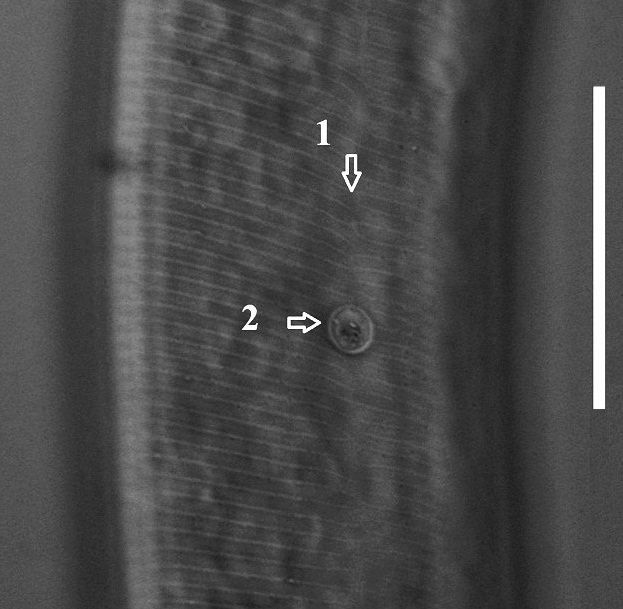
Posterior phasmid position (arrow 2) and lateral field in lateral view (arrow 1)

**Figure 1f.**
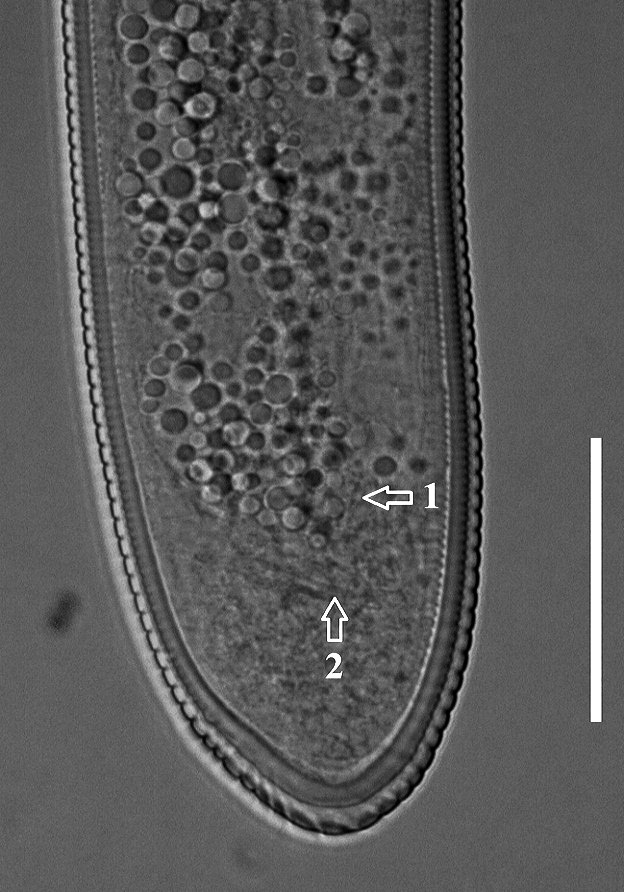
Posterior end, intestine (arrow 1) and rectum (arrow 2)

**Figure 2a.**
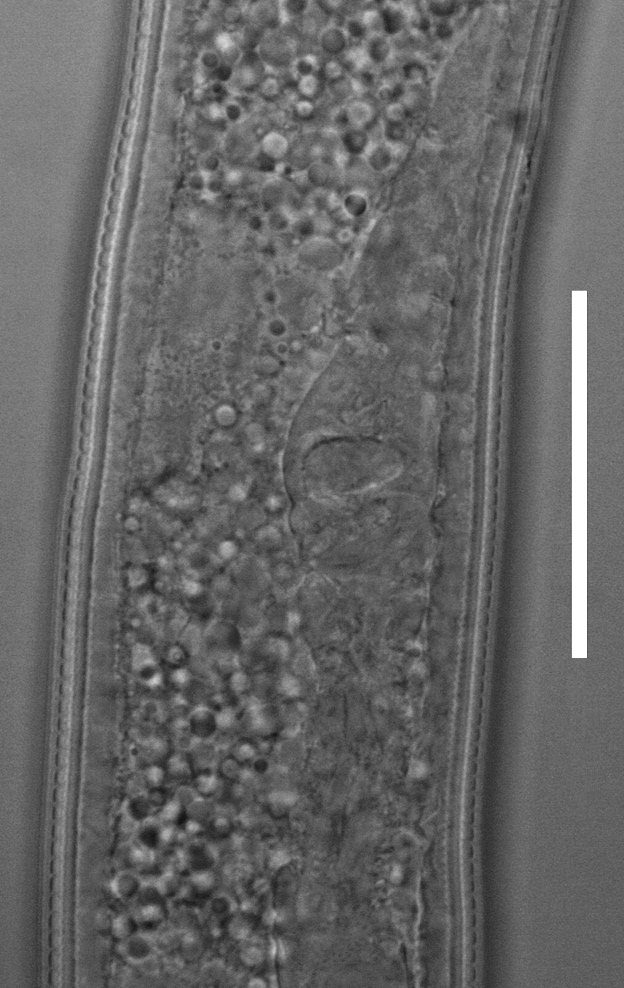
Spermatheca

**Figure 2b.**
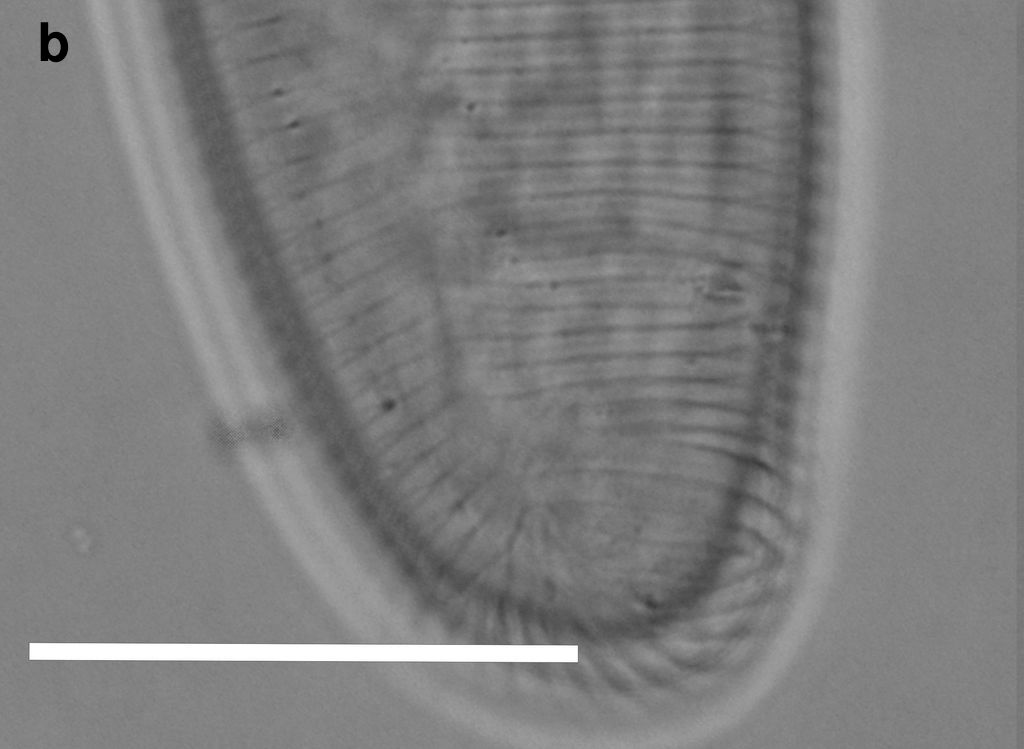
Posterior end in ventrosublateral view

**Figure 3a.**
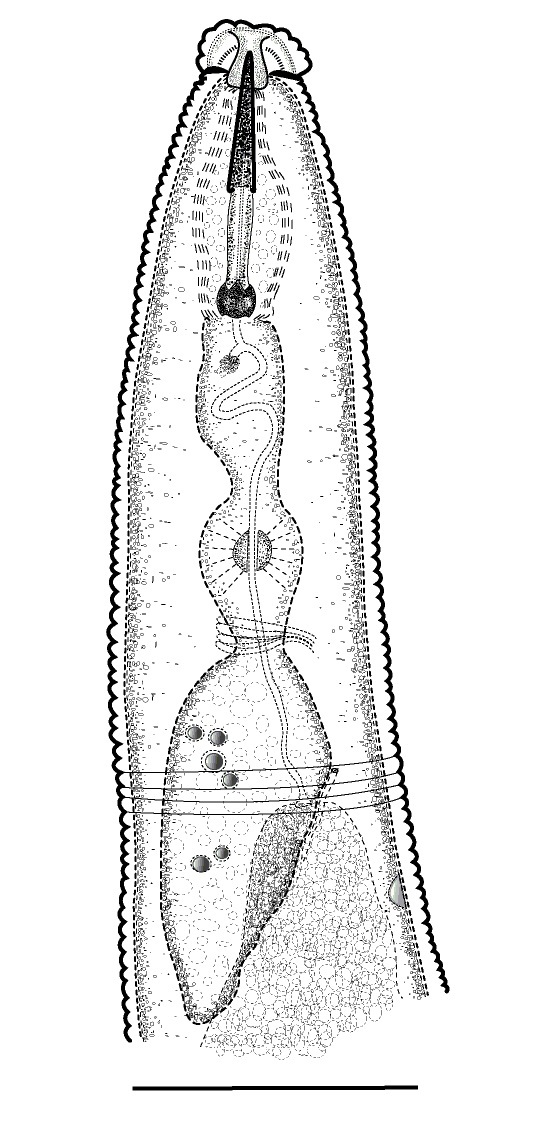
Anterior end

**Figure 3b.**
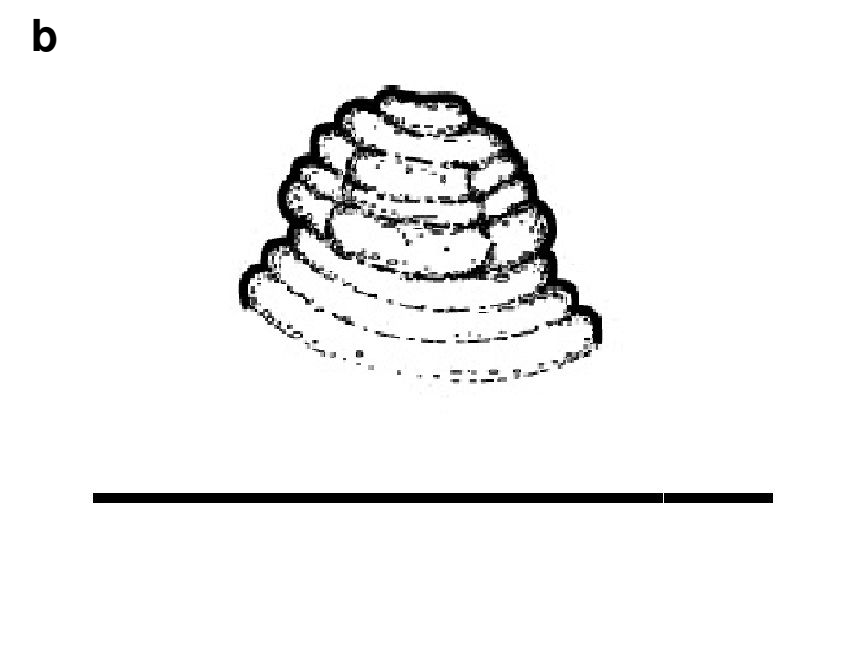
Lip region

**Figure 3c.**
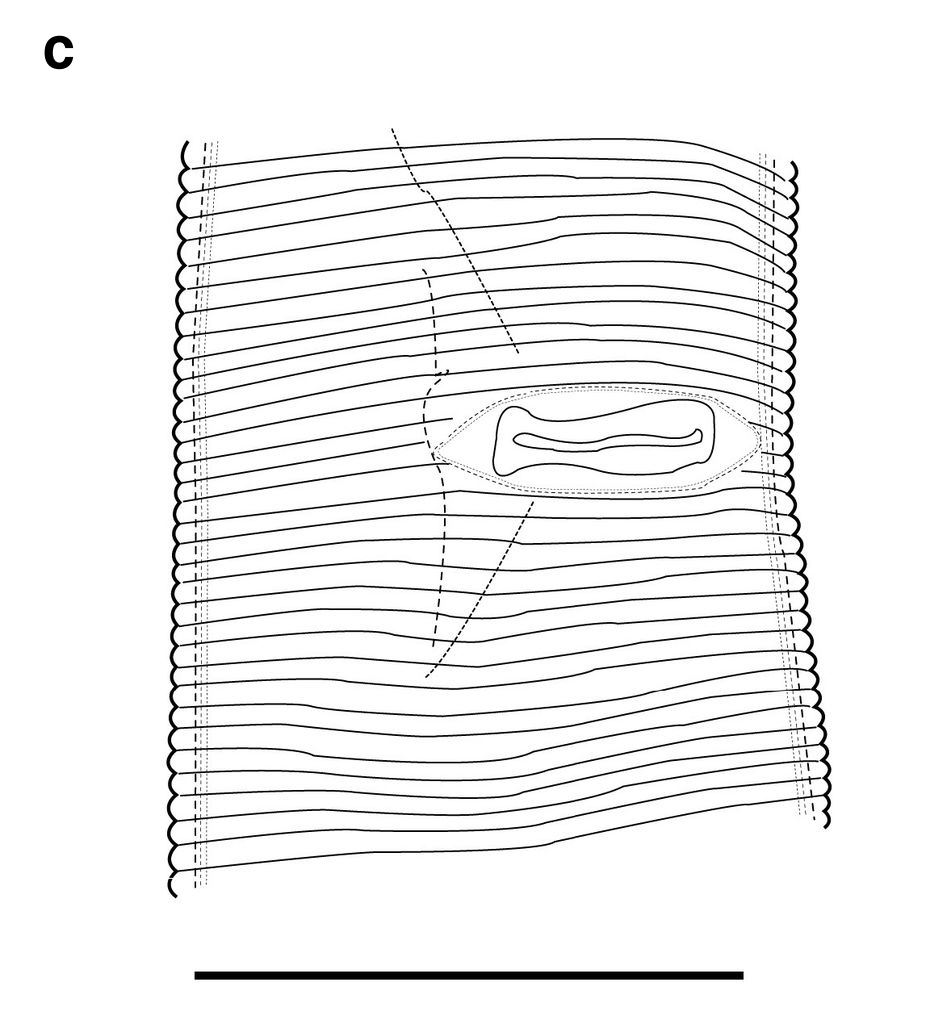
Vulva region (ventral view)

**Figure 3d.**
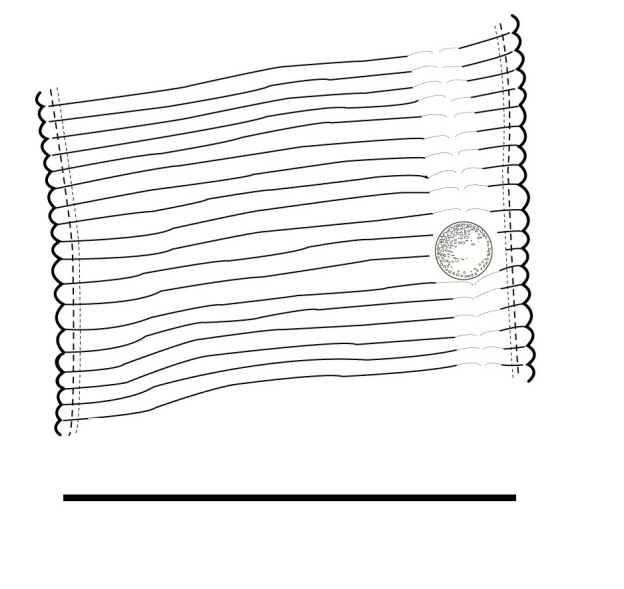
Posterior phasmid.

**Figure 3e.**
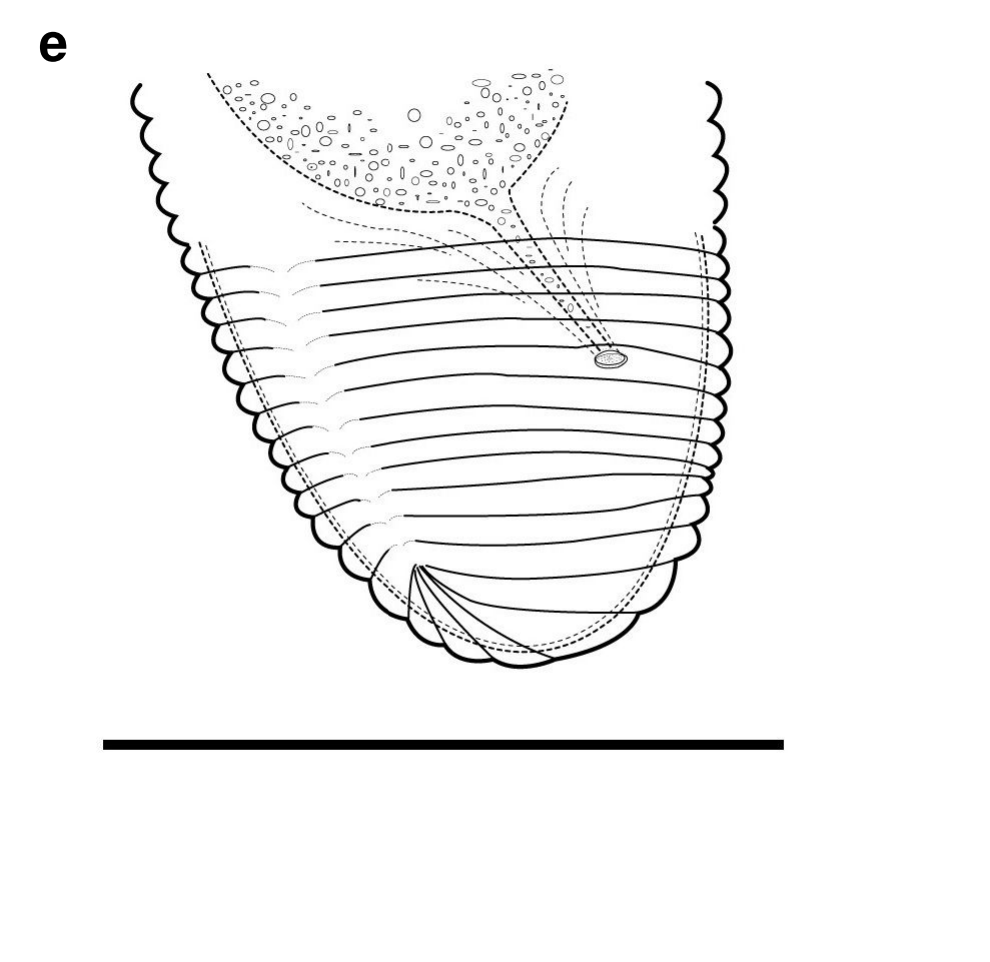
Posterior end (ventrosublateral view)

**Figure 3f.**
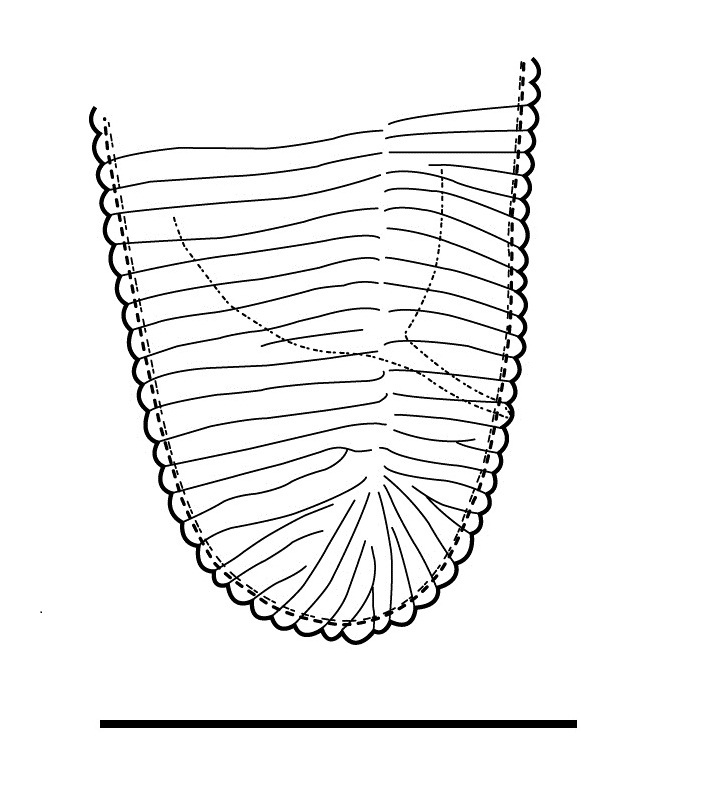
Posterior end (lateral view)

**Table 1.** Morphometrics of *Hoplolaimus bachlongviensis* sp. n. (all measurements in µm, as mean ± standard deviation (range).

**Measurements**	**Holotype female**	**Paratype females**
n	1	8
Body length	1439	1405 ±78.2 (1247-1493)
Stylet cone length	26	25.5±1.1 (24-27)
Stylet knob length	7	7.2±0.7 (6-8)
Stylet length	50	47.1±2.2 (44-50)
Lip region height	9	8.8±0.6 (8-9)
Lip region diam.	18	18.1±1.0 (17-20)
Anterior end to nerve ring	147	126.5±11.9 (108-147)
Anterior end to excretory pore	154	144.2±8.6 (131-154)
Anterior end to end of pharyngeal glands	248	207.5±22.2 (175-248)
DGO	6	4.2±1.2 (3-6)
Anterior end to intestine-pharyngeal valve	170	154.6±13.2 (137-174)
Anterior phasmid of body length (%)	34	34.7±4 (29-38)
Posterior phasmidof body length (%)	80	78.6±4.5 (74-84)
Max body diam.	58	58.0±4.2 (51-66)
a	24.7	24.3±1.6 (22-27)
b	5.8	6.8±0.6 (6-8)
c	53.2	55.9±5.1 (48-64)
c’	0.7	0.7±0.1 (0.6-0.8)
V	56	56.7±1.7 (53-59)
Anal body diam.	33	36.1±2.3 (33-40)
Tail length	27	25.2±1.6 (23-27)
Tail annules	13	11 ±1.6 (9-13)
